# Co-delivery of Gefitinib and chloroquine by chitosan nanoparticles for overcoming the drug acquired resistance

**DOI:** 10.1186/s12951-015-0121-5

**Published:** 2015-09-22

**Authors:** Liang Zhao, Guang Yang, Yijie Shi, Chang Su, Jin Chang

**Affiliations:** School of Materials Science and Engineering, School of Life Sciences, Tianjin University, Tianjin, 300072 People’s Republic of China; School of Pharmacy, Liaoning Medical University, Jinzhou, 121000 People’s Republic of China; Department of Oncology, BenQ Medical Center, Nanjing Medical University, Nanjing, 210029 People’s Republic of China; School of Veterinary Medicine, Liaoning Medical University, Jinzhou, 121000 People’s Republic of China

**Keywords:** Acquired resistance, Nanoparticles, Gefitinib, Chloroquine, Autophagy

## Abstract

**Background:**

Acquired drug resistance is becoming common during cancer chemotherapy and leads to treatment failure in clinic. To conquer acquired drug resistance, nanotechnology has been employed to deliver drug. In this paper, we prepared chitosan nanoparticles (CS NPs) capable of entrapping Gefitinib and chloroquine (CQ) for multiple drugs combinational therapy.

**Results:**

The results showed that Gefitinib/CQ-NPs were characterized of small particle size about 80.8 ± 9.7 nm and positive zeta potential about 21.3 ± 1.56 mV, and drug controlled to release slowly on a biphasic pattern. Compared with free Gefitinib and Gefitinib loaded NPs, Gefitinib and CQ co-delivery by CS nanoparticles showed the higher inhibition rates and enhanced cell apoptosis. Through western blot analysis, we found that Gefitinib could promote LC3 expression, which is the marker of autophagosomes. So, the acquired drug resistance may be associated with autophagy. CQ as an inhibitor of autophagolysosomes formation could overcome autophagy in the resistant cells.

**Conclusions:**

These findings demonstrated that chitosan nanoparticles entrapping Gefitinib and chloroquine have the potential to overcome acquired resistance and improve cancer treatment efficacy, especially towards resistant strains.

**Graphical abstract:**
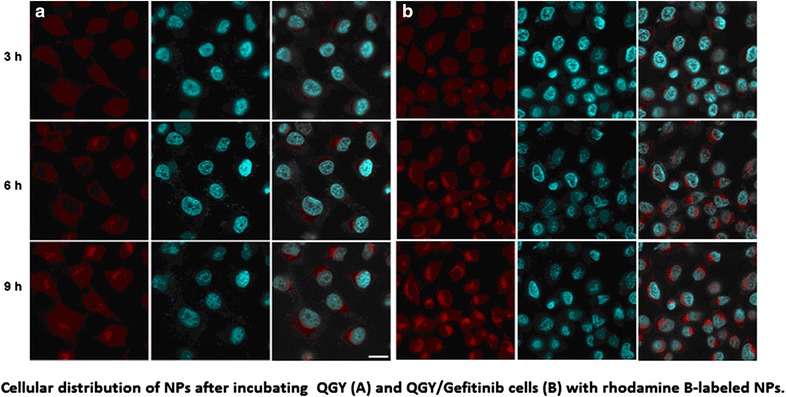
Cellular distribution of NPs after incubating QGY (a) and QGY/Gefitinib cells (b) with rhodamine B-labeled NPs.

## Background

Tyrosine kinase inhibitors targeting epithelial growth factor receptor (EGFR) have achieved great success in the treatment of malignant tumors [1–4]. Application of EGFR inhibitors to cancer patients with EGFR mutations will effectively alleviate the symptoms of pain, inhibit tumor metastasis, and even kill cancer cells. However, unfortunately, after a time period of drug exposure, the treatment response to chemotherapy in some patients will be highly declined due to a effect known as acquired resistance [5, 6]. Although many new therapeutic strategies have been applied to solve this problem, acquired resistance occurred frequently in clinical cancer therapy and resulted in chemotherapy failure.

Autophagy is the basic catabolic mechanism that involves cell degradation of unnecessary or dysfunctional cellular components through the actions of lysosomes. In the autophagic process, the autophagosome and lysosome were fused to form autophagic lysosome, degrading large or deformed molecules in cytoplasm and cellular organelles, and recycling the degraded product to provide nutritive material for the normal survival and metabolism of cells [7–9]. According to recent research, autophagy may take great part in promoting acquired resistance effects of cancer cells. Autophagy facilitates tumor cells to survive in the adverse environment by accelerating the growth of tumor cells and preventing antitumor drugs from killing cells [10–13].

Nanoparticles as a novel drug delivery system showed its potential to overcome the acquired resistance [14, 15]. Firstly, compared with traditional drug delivery system, nanoparticles are more likely to accumulate in the interior of solid tumor for a longer time due to its small particle size and thus enhance drug concentration in target tumor site and improve the therapeutic efficacy [16–19]. Secondly, nanoparticles are capable of encapsulating more drugs or different kinds of drugs at the same time, thus efficiently protecting drugs from being degraded. Especially, drug-release can be accurately adjusted by choosing different biomaterials and controlling the structure change of nanoparticles based on the clinical requirement of the treatment [20–22]. More importantly, in order to improve target delivery of anti-cancer drugs, specific targeting molecules (such as monoclonal antibodies) can be conjugated at the surface of nanoparticles to achieve active targeting effect by binding of these molecules with specific cell surface receptors. This will reduce the total dose of drugs and thus lower the toxicity [23–26].

In this study, chitosan nanoparticles (CS NPs) entrapping both anti-cancer drug Gefitinib and chloroquine, an known inhibitor of autophagolysosomes formation, were prepared and their ability to enhance delivery of anticancer drugs against tumor acquired resistance was determined. The results showed that nanoparticles with good spherical monodispersity and positive surface could greatly facilitate Gefitinib uptake and enhance the toxicity in QGY/Gefitinib cells (established Gefitinib resistant). Gefitinib and CQ co-delivery by CS nanoparticles significantly enhanced apoptosis of QGY/Gefitinib cells by inhibiting autophagy. Western blot analysis also confirmed that expression of caspase-3 protein as the main apoptosis relevant protein was elevated and the ratio of LC3II to LC3I as an autophagosome marker was decreased. Our results demonstrated that the anticancer activity of Gefitinib can be enhanced by inhibition of autophagy in Gefitinib resistant QGY cells. These findings suggested that simultaneous delivery of Gefitinib and CQ by CS nanoparticles might be a promising treatment to reverse acquired resistance in tumor cells.

## Results and discussion

### Physicochemical properties of NPs

**Fig. 1:**
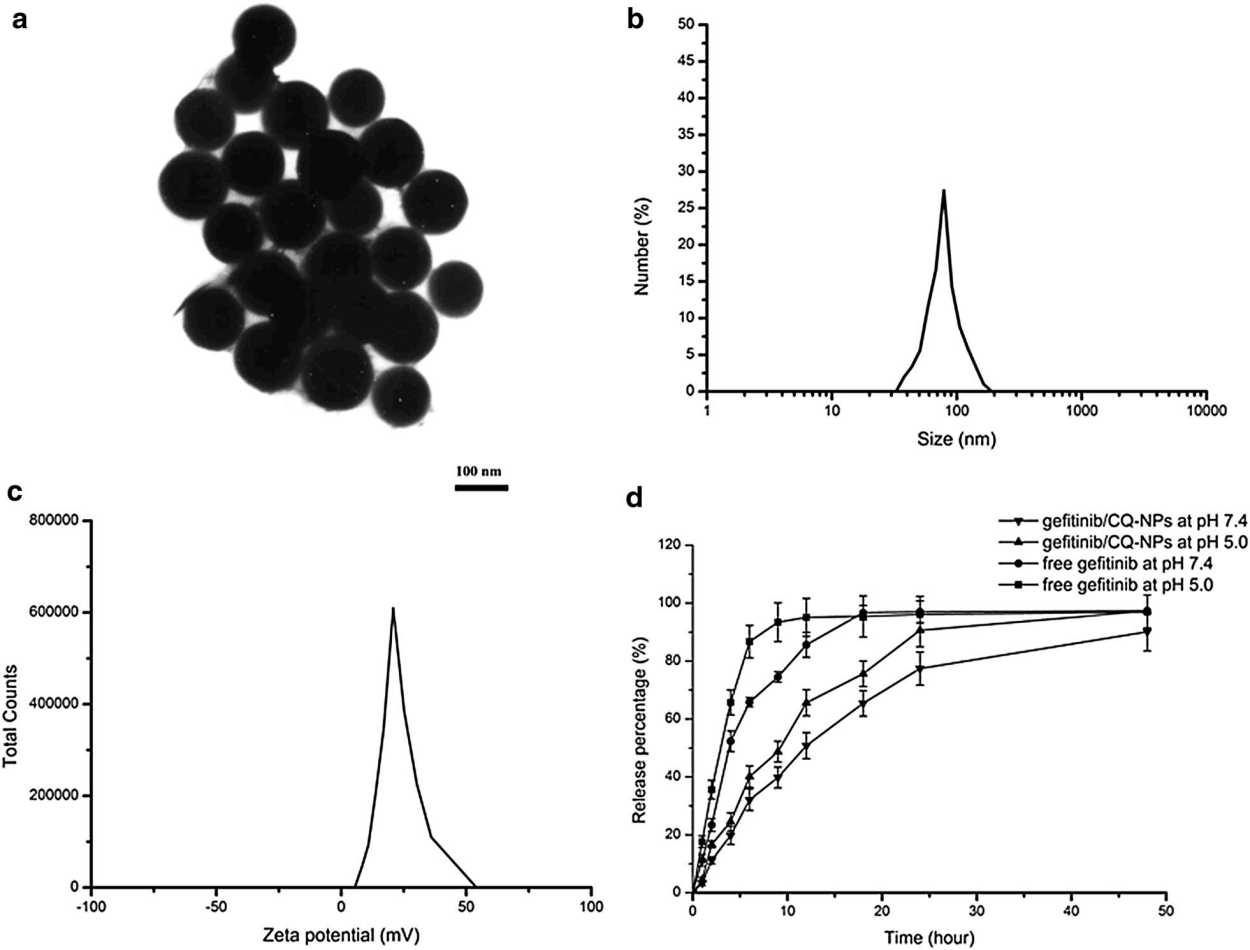
Characterization of Gefitinib/CQ-NPs. **a** TEM images of Gefitinib/CQ-NPs, **b** DLS analysis of Gefitinib/CQ-NPs, **c** zeta potential of Gefitinib/CQ-NPs, and **d** accumulative release of Gefitinib/CQ-NPs and free Gefitinib in the medium at different pH. The results were expressed as mean ± SD (n = 3)

The morphology and size distribution of the prepared nanoparticles were determined by means of TEM and DLS. As shown in Fig. 1, Gefitinib/CQ-NPs were characterized of small particle size about 80.8 ± 9.7 nm and positive zeta potential about 21.3 ± 1.56 mV. In addition, the polydispersity index of Gefitinib/CQ-NPs was above 0.023, indicating the homogenous monodispersion and good stability in media. As for the encapsulation efficiency of drugs, about 80.7 ± 3.4 % of Gefitinib and 78.6 ± 4.6 % of total CQ were encapsulated in NPs. In term of drug release in vitro, Gefitinib/CQ-NPs and free Gefitinib at the same concentration were loaded in dialysis bags with molecular weight cutoff of 1000 and immersed in 60 mL of PBS, and the aliquots from release media were taken out after certain time intervals for determining the release pattern of Gefitinib in media. The results showed that compared with the fast release of free drug, drugs encapsulated in NPs could be controlled to release slowly and smoothly at different pH. Generally, Gefitinib/CQ-NPs reflected biphasic drug release pattern with a burst release within the initial 2 h and a sustained release afterwards. With the decrease of pH, the releasing rate of drug was increased and more drugs diffused out from the interior of NPs. It possibly suggested that the total drug-release amount from the NPs depended on the drug solubility and the penetration from the core of NPs. In the acidic condition, the amine groups from chitosan can be bond with H^+^ to form cationic polyelectrolytes. Due to the electrostatic repulsion between molecules of chitosan, the molecular chains of chitosan tended to extend and were easily further degraded into small parts, thus facilitating drugs to diffuse into the media.

### In vitro cellular uptake

**Fig. 2:**
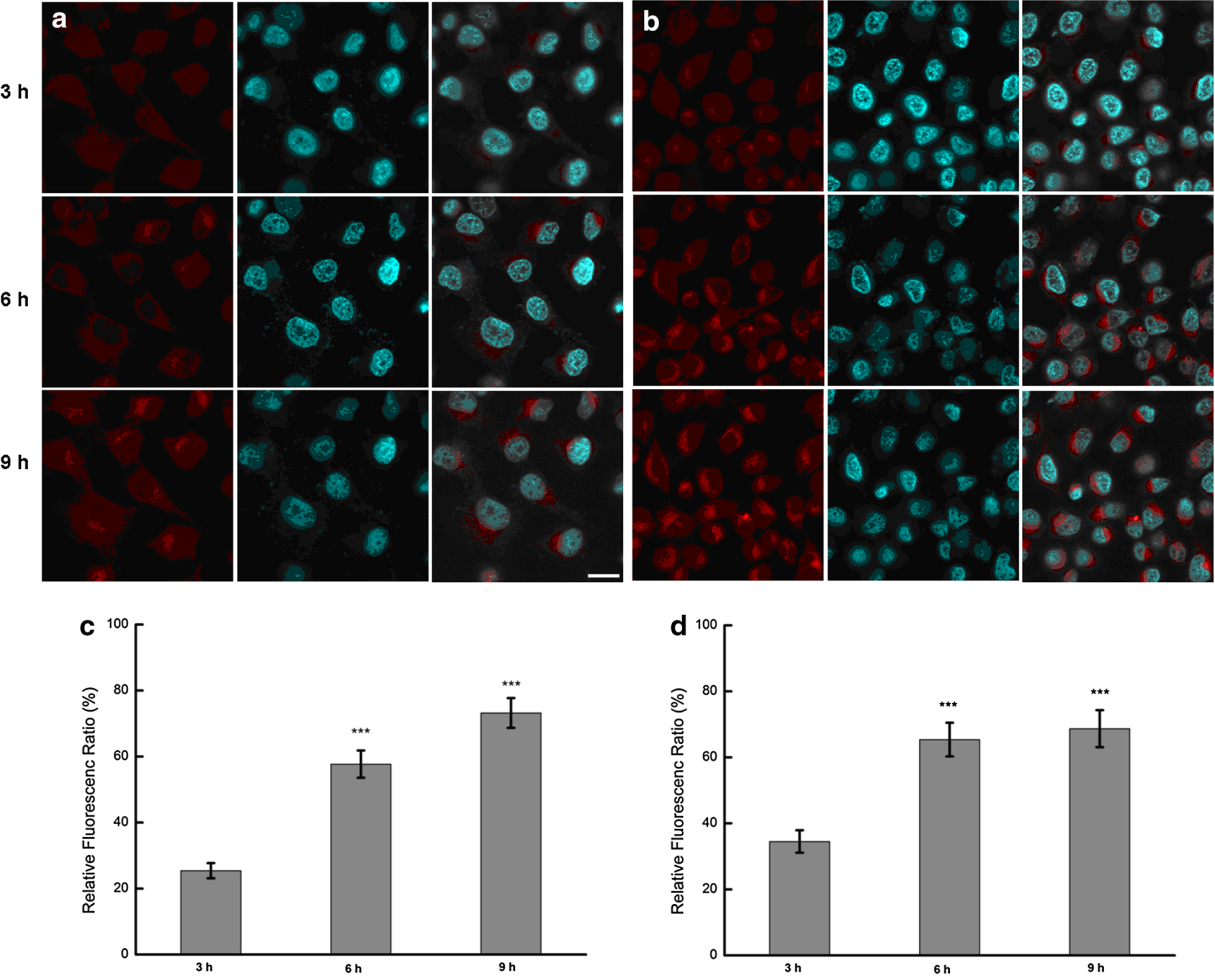
In vitro cellular distribution of NPs after incubating different tumor cells with rhodamine B-labeled NPs. Fluorescent image of QGY (**a**) and QGY/Gefitinib cells (**b**), the scale bar is 50 μm, fluorescence spectrum analysis of QGY (**c**) and QGY/Gefitinib cells (**d**)

The cell uptake of NPs in QGY and QGY/Gefitinib cells were observed as shown in Fig. 2. Rhodamine B as the fluorescent marker was encapsulated in NPs and the cell nucleus was stained with Hochest (blue) for 15 min at 37 °C. The results demonstrated that within the initial 3 h, some weak red fluorescence was found around the cells, indicating the gradual aggregation and disperse of NPs towards cells. With the extension of time, the intracellular red fluorescence became stronger and NPs could be sprinkled throughout the whole cytoplasm. The cellular distribution of NPs was quantified by micro-plate reader and is shown in Fig. 2. When NPs were incubated with the two tumor cells for 9 h, The RFR observed in cells was increased gradually from about 30 % in the initial 3 h to over 60 % at 9 h. It suggested that NPs showed the similar intracellular uptake pattern in two cells and the internalization of nanoparticles into the cell was time dependent. Due to electrostatic attraction, CS NPs with positive charges tended to combine with negatively charged cell surface protein to form clathrin vesicles. When vesicles fell off from the membrane into the cytosol, they fused with endosomes or lysosomes to form a collection. And in the acidic environment, these enzymes could break chemical bonds and particle structure, thus leading to the rapid release of drugs.

### Uptake mechanisms involved in the cellular entry of NPs

**Fig. 3:**
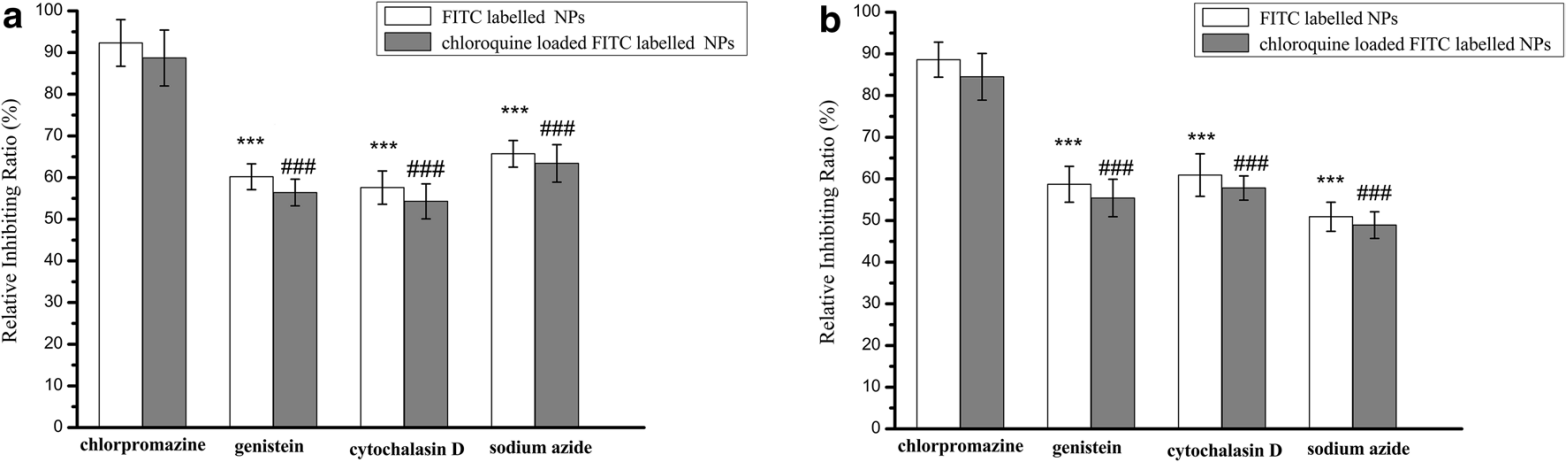
Study on uptake mechanisms involved in the cellular entry of NPs. **a** QGY cells. ***P < 0.001, vs FITC-labeled NPs treated with chlorpromazine. ^###^P < 0.001, vs the chloroquine loaded FITC-labeled NPs treated with chlorpromazine. **b** QGY/Gefitinib cells. ***P < 0.001, FITC-labeled NPs treated with chlorpromazine. ^###^P < 0.001, vs the chloroquine loaded FITC-labeled NPs treated with chlorpromazine. Results were expressed as mean ± SD (n = 3)

Location and distribution of NPs in cells depended on uptake process and NPs could be internalized into cells mainly through the following several endocytic pathways including caveolae-mediated endocytosis, macropinocytosis and clathrin-mediated endocytosis. In order to further elucidate the internalizing process of NPs in cells, cells were preincubated with some inhibitors to block different endocytic pathways followed by the addition of NPs. After continuous treatment with NPs for 12 h, cells in well were washed with ice-cold PBS for three times to remove the NPs outside cells and the internalization efficiency of NPs in cells was quantified by measuring the ratio of the fluorescent intensity of internalized FITC-labeled NPs to that from the initially added FITC-labeled NPs, expressed as a percentage. The results shown in Fig. 3 demonstrated that when pretreated with genistein as an inhibitor of caveolae-mediated endocytosis, the internalization efficiency of NPs in both cells was significant reduced. Compared to that in untreated cells, the uptake of NPs and chloroquine loaded NPs in QGY cells pretreated with genistein was reduced to 60.2 and 56.4 %, while the uptake in QGY/Gefitinib cells was 58.7 and 55.4 %. After treated with cytochalasin D for blocking macropinocytosis, the uptake efficiency of NPs was reduced to 57.6 % in QGY cells and 60.9 % in QGY/Gefitinib cells, and in comparison, the intracellular uptake of chloroquine loaded NPs was reduced to 54.3 % in QGY cells and 57.8 % in QGY/Gefitinib cells. Especially, with the addition of sodium azide as an energy inhibitor for decreasing intracellular ATP generation, internalization of both NPs in cells was significant lower than that in untreated cells (65.7 % for NPs and 63.4 % for chloroquine loaded NPs in QGY cells, and 50.9 and 48.9 % in QGY/Gefitinib cells). On the contrast, the regulation of clathrin-mediated endocytosis on NPs internalization was not significantly different from untreated group. The uptake of NPs and chloroquine loaded NPs in QGY cells pretreated with chlorpromazine were 92.3 and 88.7 % in QGY cells, 88.6 and 84.5 % in QGY/Gefitinib cells. The results suggested that the endocytosis of NPs in cells was energy dependent and mainly relied on caveolae-mediated endocytosis and macropinocytosis uptake. It also demonstrated that involvement of CQ as an inhibitor of autophagy seemed to have no significant difference in NPs internalization suggesting a minor role of autophagy on the intracellular internalization process of NPs.

### MTT assay

**Fig. 4:**
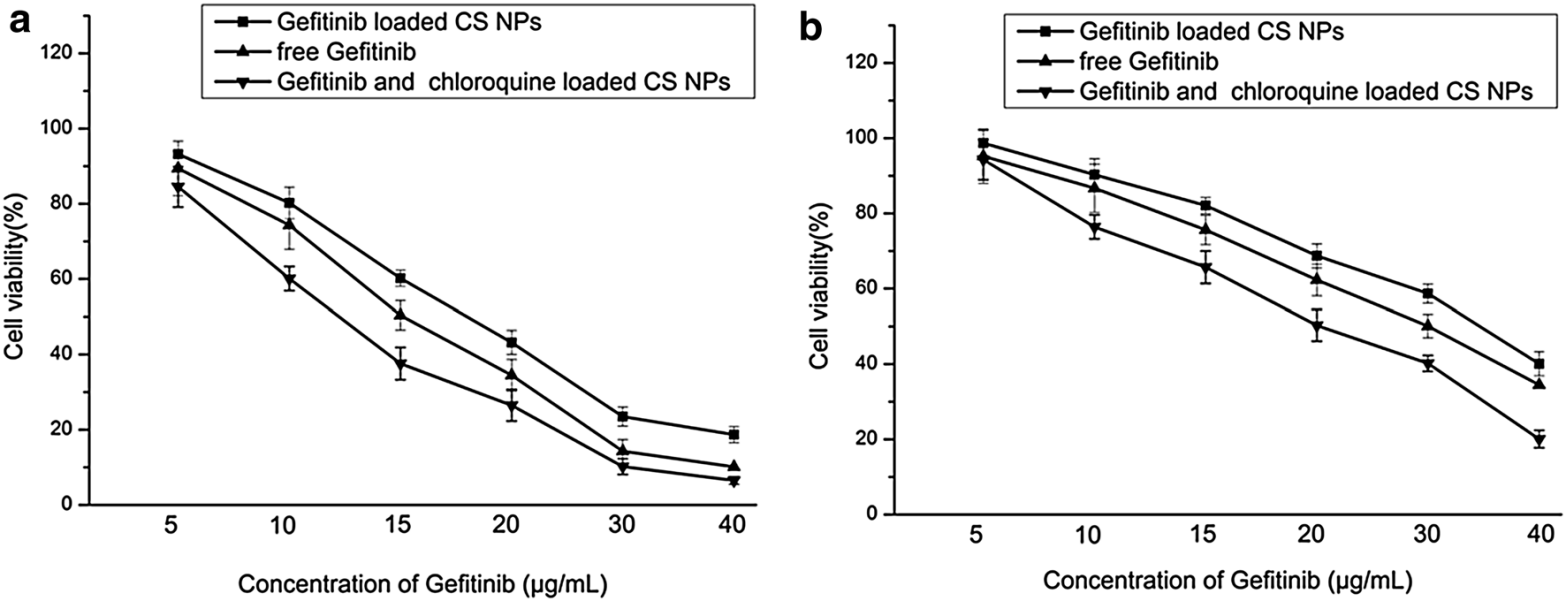
The results of cell viability assays. **a** Viability of QGY cells after incubating with different amounts of free Gefitinib, Gefitinib loaded NPs and Gefitinib/CQ-NPs for 48 h (n = 3). **b** Viability of QGY/Gefitinib cells after incubating with different amounts of free Gefitinib, Gefitinib loaded NPs and Gefitinib/CQ-NPs for 48 h (n = 3). Data were presented as mean ± SD (n = 3)

In order to evaluate the inhibitory effects of free drugs and drug loaded NPs in two cells, MTT assay was used to investigate cell viability and the result was shown in Fig. 4. It demonstrated that when treated with free Gefitinib, QGY/Gefitinib cells were more resistant to the treatment compared with QGY cells, and the IC50 values in free Gefitinib treated QGY and QGY/Gefitinib cells within 48 h were 15.1 and 30.1 μg/mL. Compared with free drugs, Gefitinib loaded NPs showed relative lower inhibition rates and the IC50 values in QGY and QGY/Gefitinib cells within 48 h were 18.0 and 34.7 μg/mL. Interestingly, when two cells were incubated with NPs encapsulating both Gefitinib and CQ, the cells viability was significantly reduced. The IC50 values of QGY and QGY/Gefitinib cells treated with Gefitinib/CQ-NPs were 12.2 and 20.1 μg/mL. It implied that the co-delivery of Gefitinib and CQ enhanced the cell apoptosis by down-regulating the autophagy. Autophagy can protect tumor cells from the damage of chemotherapy by clearing damaged macromolecules or mitochondria, thereby preventing tumor cells from undergoing the apoptosis.

### Cell apoptosis and necrosis

**Fig. 5:**
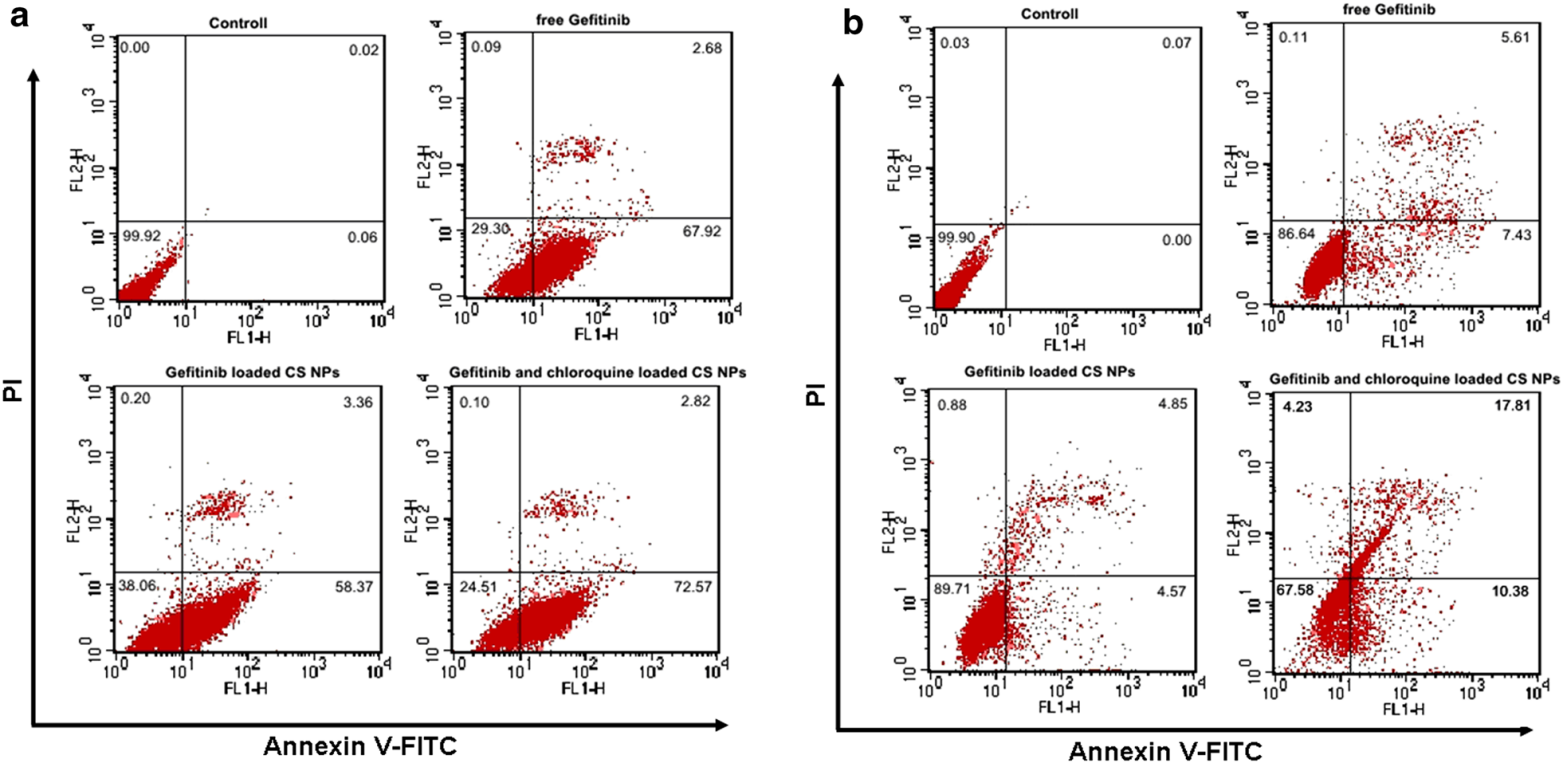
Cell apoptosis determined by Annexin V-FITC staining. Flow cytometer analysis of the apoptosis and necrosis cells after 48 h incubation with the free Gefitinib, Gefitinib loaded NPs and Gefitinib/CQ-NPs, respectively. Results were expressed as mean ± SD (n = 3). **a** QGY cells, **b** QGY/Gefitinib cells

In order to justify the role of autophagy on the cell apoptosis, Annexin V-FITC/PI staining assay was performed and the apoptotic and necrotic cells were quantified by flow cytometry. The results showed that with addition of free Gefitinib or Gefitinib loaded NPs, the apoptosis effects were enhanced more in QGY cells than QGY/Gefitinib cells. Flow cytometry analysis revealed that after 48 h treatment with free Gefitinib and Gefitinib loaded NPs in QGY cells, the ratio of AV-positive and PI positive cells in was 70.70 and 61.94 %, respectively. On the contrary, less than 20 % of QGY/Gefitinib cells underwent the apoptosis when treated with free Gefitinib or Gefitinib loaded NPs, indicating that QGY/Gefitinib cells were less sensitive to the treatment of the medicine. Interestingly, with the mediation of CQ as an inhibitor of autophagolysosomes formation, lysosomal acidification was inhibited and therefore prevented autophagy by blocking autophagosome fusion and degradation. The ratio of AV-positive and PI positive cells treated with the Gefitinib/CQ-NPs was obviously increased. Especially for QGY/Gefitinib cells, compared with low ratio of AV-positive and PI positive cells after the treatment of free drugs (about 13.36 %) and Gefitinib loaded NPs (about 10.29 %), the ratio of AV-positive and PI positive fractions in Gefitinib and CQ loaded NPs treated QGY/Gefitinib cells was 32.42 %, markedly higher than other treatments. It demonstrated that cell autophagy can serve as a defense tool to protect cells from damaging and death due to the environment changes. The results suggested that autophagy can adversely affect apoptosis, and blockade of autophagy will increase the sensitivity of cells to apoptosis signal (Fig. 5).

### Western blot analysis

**Fig. 6:**
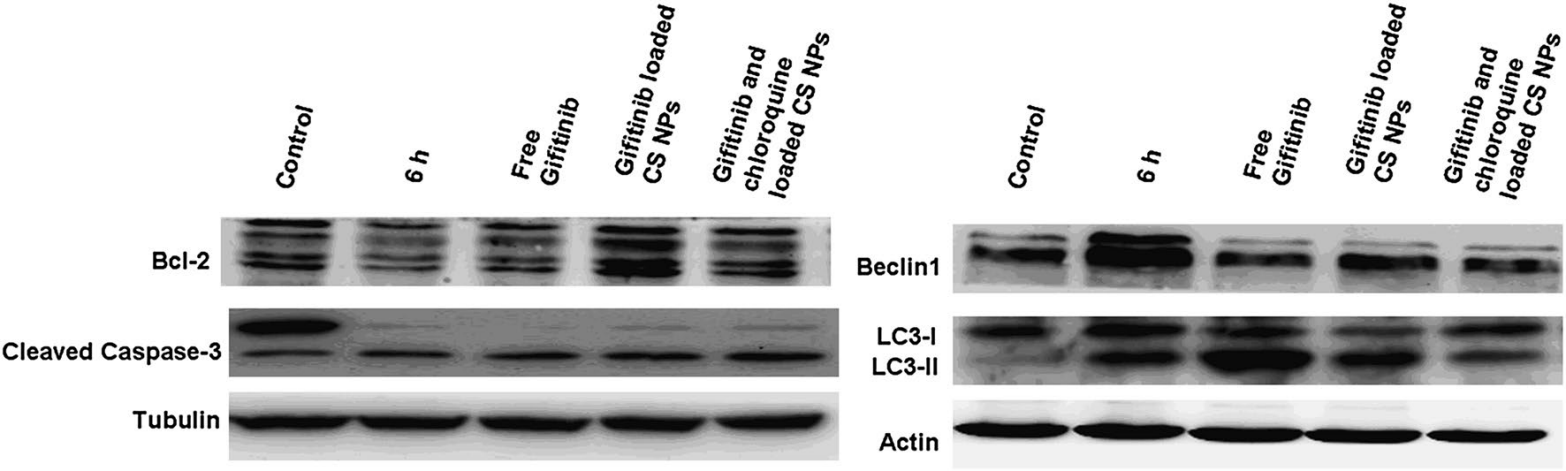
Apoptotic effects of various Gefitinib formulations on QGY/Gefitinib cells after treatments

To identify the effect of Gefitinib and Gefitinib loaded NPs on cell apoptosis and autophagy, we detected the expression of caspase-3, Bcl-2, Beclin1 and LC3 by western blot experiment. Compared with free Gefitinib and Gefitinib loaded NPs, Gefitinib/CQ-NPs induced the highest caspase-3 protein expression. This indicated that co-delivery of CQ and Gefitinib encapsulated in NPs might significantly enhance the apoptosis represented by the increasing expression of caspase-3 as the main apoptosis relevant protein in western blot. Western blot analysis also suggested that after 6 h of hungry treatment in QGY/Gefitinib cells, the ratio of LC3 II to LC3 I was increased, implying the accumulation of autophagic vesicles in cells and therefore autophagy induction. This could be explained as a response to cell stress produced due to the change of the culture conditions. Similarly, free Gefitinib and Gefitinib loaded NPs also enhance the autophagy by the augment of LC3 II levels. Chloroquine as a lysosomal targeting drug, can enter the lysosome selectively, and inhibit phospholipid A2 and the release of arachidonic acid, and then further prevent autophagy through cytoplasmic degradation by lysosome. The results demonstrated that co-delivery of CQ and Gefitinib encapsulated in NPs significantly inhibited the autophagy effects by down-regulating the ratio of LC3 II and LC3 I, thus increasing the sensitivity of QGY resistant cells to Gefitinib and promoting the apoptosis of cells through the increasing expression of caspase-3 proteins. Interestingly, Beclin1 and apoptosis inhibition factor Bcl-2 in cells treated with free drug and drug loaded NPs showed a negative correlation on expression level. When cells were incubated under the starving conditions for 6 h, the expression of Bcl-2 was reduced while the beclin1 protein was highly expressed. With the addition of free drugs or drug loaded NPs into cells, Bcl-2 protein expression was enhanced and the expression level of beclin1 was inhibited. It suggested that the former may down-regulate the expression of the latter to prevent the development and progression of autophagy (Fig. 6).

### Intracellular ATP level assay

**Fig. 7:**
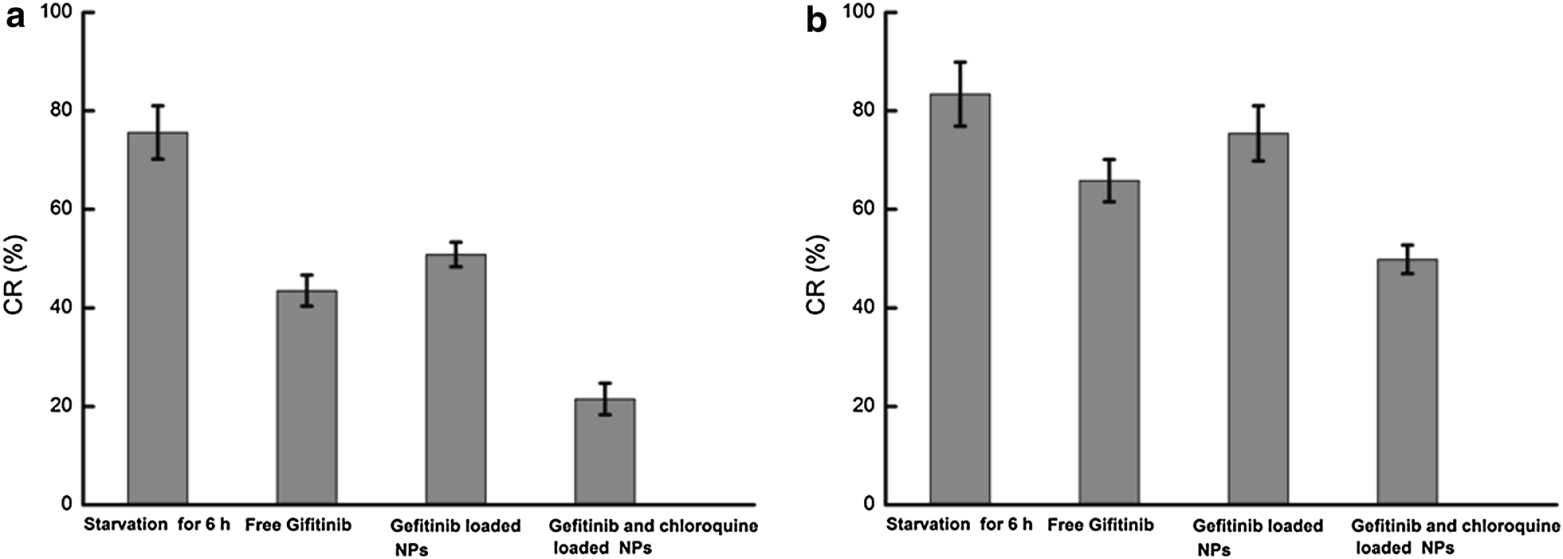
Intracellular ATP level assay after 48 h incubation with different Gefitinib formulations, respectively. Results were expressed as mean ± SD (n = 3). **a** QGY cells, **b** QGY/Gefitinib cells

Tumor cells are dependent on gaining ATP to maintain the cellular cleavage and proliferation. When ATP was depleted, the permeability of mitochondrion in tumor cell was enhanced and some apoptosis proteins such as cytochrome *c* (Cyt-c) and activating caspase were released, thus inducing the cell apoptosis. The results as shown in Fig. 7 demonstrated that the reduction of ATP was positively correlated with the apoptosis effects and drugs or drugs loaded NPs could induce more reduction of ATP accompanied by noticeable apoptosis in QGY cells than in QGY/Gefitinib cells. Especially, when CQ and Gefitinib were co-delivered into cells, the level of ATP was lowest and more apoptosis effects were induced. This indicated that autophagy also played an important role on the consumption and production of ATP. When cells were treated with drugs or starvation, P-AMPK can inhibit mTOR and P53 signal pathway and induce autophagy to prevent the growth and proliferation of cells, and therefore reduce the consumption of ATP. Taken together, inhibition of autophagy through the mediation of CQ in some extent accelerated the decrease of ATP level.

## Conclusions

Briefly, we found that CS nanoparticles with the co-delivery of Gefitinib and CQ enhanced delivery of anticancer drugs against tumor acquired resistance. It demonstrated that compared with free Gefitinib or Gefitinib loaded NPs, co-delivery of Gefitinib and CQ induced more apoptosis effects and the treatment response to chemotherapy was significantly improved in drug resistant cell lines. Western blot result further confirmed that with the mediation of CQ, autophagy effect was inhibited significantly by down-regulating the ratio of LC3 II and LC3 I, and the apoptosis was significantly promoted represented by the increasing expression of caspase-3 as the main apoptosis relevant protein in western blot. Taken together, it demonstrated that CS nanoparticles with the co-delivery of Gefitinib and CQ could help to overcome tumor acquired resistance in drug resistant cell lines and provided a promising combined therapeutic strategy for enhanced antitumor therapy.

## Methods

### Materials

Chitosan (CS) of medium molecular weight (deacetylation degree, 80 %; molecular weight, 400,000) was purchased from Haixin Biological Product Co., Ltd (China). Gefitinib was purchased from Eastbang Pharmaceutical Co., Ltd (China). Chloroquine, acetic acid and sodium tripolyphosphate were obtained from Sigma (St Louis, USA). All other chemicals were of reagent grade and were used as received.

### Preparation of Gefitinib/CQ-NPs

A 0.5 mg/mL CS solution was prepared by dissolving 0.25 g of chitosan in 500 mL of acetic acid (2 %, v/v) followed by the addition of sodium hydroxide solution (20 wt%) to adjust the pH of CS solution to 4.7. After that, 5 mL stock solution of Gefitinib and chloroquine at a certain concentration was added into the CS solution to obtain the mixture of drugs and CS. To prepare the Gefitinib/CQ-NPs, sodium tripolyphosphate (TPP) reserve liquid (0.5 mg/mL) was dripped slowly into CS solution under stirring until the opalescent color in solution appeared. After continuously stirring for 1 h and centrifugation at 16,000 rpm for 20 min, nanoparticles were separated and washed with distilled water for three times. Finally, the Gefitinib/CQ NPs were freeze-dried under vacuum for further analysis. To evaluate the physical characterization of Gefitinib/CQ-NPs, transmission electron microscope (TEM) (JEM-1200EX, Tokyo, Japan) and Zetasizer (Nano ZS90, Malvern, UK) were used to determine its morphology, mean diameter and zeta potential. The encapsulation efficiency (EE) of Gefitinib in NPs was calculated according to the protocol in our previous study [27]. The in vitro drug release from NPs was estimated by previously reported method [28].

### Cell culture

Hepatocellular carcinomacellline QGY cells and QGY/Gefitinib cells (established Gefitinib resistant) were purchased from the Type Culture Collection of Chinese Academy of Science (CAS). The cells were propagated in DMEM supplemented with 10 % fetal bovine serum (FBS), 2 mM glutamine, 100 U/mL penicillin and 100 μg/mL streptomycin at 37 °C, 5 % CO_2_ and passaged every 3–5 days.

### In vitro cellular uptake

In order to detect the cellular distributions of NPs, rhodamine B or fluorescein isothiocyanate (FITC) was encapsulated into NPs as a fluorescent marker and the fluorescence was observed by confocal laser scanning microscopy (FluoView FV10i, Olympus, Japan) and quantified by microplate reader (Synery-2, Biotek, USA). Briefly, confluent QGY cells and QGY/Gefitinib cells were seeded in the 6-well under incubation to reach a density of 5 × 10^4^/mL. After 24 h, rhodamine B-labeled NPs were added into the medium and cells in well were washed with ice-cold PBS for 3 times to remove the NPs that was not internalized in cells at the predetermined interval. Finally, cells treated with rhodamine B-labeled NPs were collected and the internalization of the rhodamine B-labeled NPs was observed using confocal laser scanning microscopy. The internalization efficiency of NPs in cells was quantified by measuring relative fluorescent ratio (RFR) of the fluorescent intensity of internalized FITC-labeled NPs to that from the initially added FITC-labeled NPs, expressed as a percentage [28].

### Tracking of uptake pathways using various endocytic inhibitors

In order to investigate the effect of various inhibitors on the uptake pathway of NPs. Both QGY cells and QGY/Gefitinib cells were preincubated with different inhibitors such as 10 μg/mL chlorpromazine (inhibition of clathrin-mediated uptake), 1 μg/mL genistein (caveolae-mediated uptake), cytochalasin D (30 μM, macropinocytosis) and 20 μg/mL sodium azide (an energy inhibitor) according to the protocol of our previous study [28]. The internalization efficiency of NPs in cells was quantified by measuring the ratio of the fluorescent intensity of internalized FITC-labeled NPs to that from the initially added FITC-labeled NPs represented as RFR, expressed as a percentage.

### MTT assay

MTT assay (CellTiter 96^®^ Aqueous One Solution Reagent) was used to investigate cytotoxicity of all the samples on both QGY cells and QGY/Gefitinib cells. According to the protocol of our previous study, free Gefitinib, Gefitinib-NPs and Gefitinib/CQ-NPs with the same concentration of Gefitinib were treated both cells for 48 h at 37 °C. The absorbance of the solution was measured using a microplate reader (Syneray-2, Biotek, USA) at 490 nm.

### Annexin V-FITC/PI staining by FCM

According to the protocol of our previous study [27], confluent QGY cells and QGY/Gefitinib cells were incubated with free Gefitinib, Gefitinib-NPs and Gefitinib/CQ-NPs with the same concentration of Gefitinib for 48 h at 37 °C. After that, the adherent cells were digested with trypsin without EDTA and collected by centrifugation for 5 min followed by constant washing with cold PBS twice. 100 μL of binding buffer was added into the obtained resuspended cells, and succeeded by reacting with 5 μL Annexin V-FITC and 5 μL PI staining solution under constant stirring away from light at the room temperature for 10 min. Finally, 400 μL of binding buffer was added and the apoptosis of cells was determined using the flow cytometer FACS-Calibur (Becton–Dickinson, San Jose, CA, USA).

### Intracellular ATP level assay

After being treated with free Gefitinib, Gefitinib-NPs and Gefitinib/CQ-NPs with the same concentration of Gefitinib for 48 h at 37 °C, the change of intracellular ATP level in cells was determined using the luciferrin-luciferase-based ATP luminescence assay kit. The changing rates of intracellular ATP level (CR) were evaluated by calculating the ratio of ATP level in cells treated with free drug or NPs to ATP level of untreated cells.

### Western Blot assay

Free Gefitinib, Gefitinib-NPs and Gefitinib/CQ-NPs were incubated with QGY/Gefitinib cells for 48 h followed by washing twice with ice-cold PBS. RIPA buffer (150 mM NaCl, 1 % NP-40, 1 % SDS, 1 mM PMSF, 10 µg/mL leupeptin, 1 mM aprotinin, 50 mM Tris–Cl, pH 7.4) was used for lysis and removed by centrifugation at 12,000 rpm for 25 min. Next, cell lysate was separated using 10 % SDS-PAGE and the protein was transferred onto polyvinylidene fluoride (PVDF) membrane. After blocking of non-specific binding by placing the membrane in a dilute solution of 1 % BSA, the membrane was incubated with the primary antibody at 4 °C overnight and appropriate secondary antibody for 1 h, respectively. Finally, the level of the targeted proteins was photographed and analyzed by UVP gel analysis system.

## Abbreviations

CS NPs: chitosan nanoparticles
CQ: chloroquine
